# Community-based Mobile Cervical Cancer Screening Program in Rural India: Successes and Challenges for Implementation

**DOI:** 10.31557/APJCP.2021.22.5.1393

**Published:** 2021-05

**Authors:** Vijaya Srinivas, Sasha Herbst De Cortina, Holly Nishimura, Karl Krupp, Poornima Jayakrishna, Kavitha Ravi, Anisa Khan, SubbaRao V. Madhunapantula, Purnima Madhivanan

**Affiliations:** 1 *Public Health Research Institute of India, Mysore, India. *; 2 *School of Medicine, University of California Irvine, Irvine, USA. *; 3 *Department of International Health, Bloomberg School of Public Health, Johns Hopkins University, USA. *; 4 *Department of Health Promotion Sciences, Mel & Enid Zuckerman College of Public Health, University of Arizona, Tucson, USA. *; 5 *Department of Biochemistry, JSS Medical College, Leader, Special Interest Group (SIG) in Cancer Biology and Cancer Stem Cells JSS Academy of Higher Education &amp; Research, Mysore, Karnataka, India. *; 6 *Division of Infectious Diseases, College of Medicine, University of Arizona, Tucson, USA. *; 7 *Department of Family & Community Medicine, College of Medicine, University of Arizona, Tucson, USA. *

**Keywords:** Cancer screening, cervical cancer, community outreach, mobile clinic

## Abstract

**Background:**

The aim of this study is to demonstrate the feasibility; mention the challenges encountered and highlight the success of implementing a community-based mobile cervical cancer-screening program in rural India.

**Methods:**

Communities were mobilized through extensive peer education and by screening in existing community spaces using a mobile clinic model. An initial “screen and treat” protocol was transitioned to “screen, test, and treat” using Pap smears for confirmatory testing, and cryotherapy or Loop Electrosurgical Excision Procedure (LEEP) for treatment. We trained 50 Peer Educators and conducted 190 screening camps in 58 locations.

**Results:**

Of 3,821 registered women, 3,544 (92.8%) accepted screening. Overall, 440/3544 (12.4%, 95% CI 11.3-13.5%) women had VIA-positive lesions. Under “screen and treat”, 56/156 (35.9%) women accepted same-day treatment. Under “screen, test, and treat”, 555/762 (72.8%) women received a Pap smear. Overall, 83 women underwent cryotherapy (n=56) and LEEP (n=27). Of those, 49 (59.0%) participants were followed up, with normal VIA results up to two years after treatment. In summary, the peer educators promoted awareness of cervical cancer and helped in gaining buy-in from communities. Acceptance of same-day treatment was low and accompanied by loss to follow-up, limiting the utility of VIA in these studies.

**Conclusions:**

Mobile infrastructure utilized in community spaces brought screening directly to rural women. Culturally appropriate methods to increase linkage to treatment and additional screening options such as HPV DNA testing should be explored.

## Introduction

Cervical cancer is the fourth most common cancer in women globally, with an estimated 569,847 new cases in 2018, and over 90% of cervical cancer deaths occurred in low- and middle-income countries (Arbyn et al., 2020). This disparity can be tied to inequalities in education, awareness, and access to screening and treatment for cervical cancer (Devarapalli et al., 2018). National screening programs have been associated with sharp declines in morbidity and mortality (Lees et al., 2016), and even a single-lifetime screening reduces mortality from cervical cancer (Sankaranarayanan et al., 2009). In India, World Health Organization (WHO) 2018 data reported 96,922 new cases and 60,078 deaths due to cervical cancer, accounting for nearly one-fifth of the global cervical cancer deaths (Arbyn et al., 2020). WHO and the Indian government’s guidelines for cervical cancer prevention recommend screening programs on the basis of resource availability(Organization, 2013; Welfare, 2016). Recommended approaches for low-resource regions include human papillomavirus (HPV) testing, visual inspection with Lugol’s iodine (VILI), and visual inspection with acetic acid (VIA). VIA is a popular method due to its low cost, instantaneous results allowing for a “screen and treat” approach, and relatively little training required, which allows for task shifting away from physicians (Mandal and Basu, 2018). Early diagnosis of pre-cancerous lesions using VIA has been successful in numerous hospital and clinic-based trials in India (Bobdey et al., 2016). 

However, India’s fourth National Family Health Survey (NFHS-4) in 2015-2016 demonstrated that only 27.5% of Indian women in rural areas had ever been screened for cervical cancer (Van Dyne et al., 2019). Rural areas of India face lower access to healthcare facilities and providers (Sharma et al., 2018). Cervical cancer prevention efforts among rural women have been further hindered by lack of awareness about HPV, cervical cancer, and cancer screening (Karunakaran et al., 2017; Patra et al., 2017). However, prior studies have shown that community-based awareness program can improve women’s knowledge and attitudes towards cervical cancer screening, that women’s groups can play a major role in promoting participation in screening programs, and that community-based education campaigns increase screening rates (Shakya et al., 2016; Musa et al., 2017).

Therefore, aligned with India’s national cancer screening program and WHO guidelines, the Public Health Research Institute of India (PHRII) developed a comprehensive cervical cancer prevention program for rural women focused on participant education, mobile infrastructure, and linkage to care. We describe the successes and lessons learned based on nine years of data from a mobile cervical cancer screening program in Mysore, India. 

## Materials and Methods

*Project site*

PHRII has been providing comprehensive reproductive healthcare for women in Mysore district since 2005. As of the most recent Indian National Census (Census, 2011), the district has a population of 3,001,127, of whom 1,489,527 are female. Approximately 58.0% of residents live in rural villages. According to NFHS-4, less than 16% of women in Mysore district had ever been screened for cervical cancer (Van Dyne et al., 2019). From July 2010 to December 2018, we conducted 190 cervical cancer screening camps in 58 rural villages and peri-urban communities. 

*Training of healthcare professionals and outreach workers*

The screening team consisted of two nurses, two counselors, four community outreach workers, three lab technicians, and three physicians. The entire team, along with an additional six gynecologists from public and private Mysore hospitals, were trained by a US-based non-governmental organization (NGO). Medical providers learned VIA, cryotherapy, and Loop Electrosurgical Excision Procedure (LEEP). Community outreach workers and counselors were trained on education and counseling using the Planning Appropriate Cervical Cancer Prevention Programs manual by PATH (Planning for Appropriate Technology in Healthcare) (Herdman et al., 2000).

*Community preparedness and outreach*

Content was adapted from the PATH manual and Where Women Have No Doctor by the Hesperian Foundation (Burns et al., 2010) to develop educational materials, and a South Indian artist created images to depict the concepts. The visuals and explanations in English and Kannada were incorporated into flipbooks suitable for educating low-literacy populations (Supplement), which were then pilot tested in the community.

PHRII collaborated with one public and two private hospitals to establish a referral system for diagnostic follow-up and treatment. Permissions were sought from the District Health Officer and Taluk (sub-district) Medical Officer to approach community health workers and carry out the program. Team members visited each community to meet with local stakeholders including leaders of NGOs and health institutions, women’s group leaders, village elders, gram panchayat (village council) leaders, and National Rural Health Mission community health workers such as Auxiliary Nurse Midwives (ANMs), Accredited Social Health Advocates (ASHAs), and Anganwadi workers. An Anganwadi is a government-run community center that is the base for maternal and child programs. Activities during these stakeholder visits included interactive awareness sessions with the flipbooks, obtaining permissions, scouting locations, and soliciting leaders’ assistance in mobilizing eligible women to attend the screening program. 

Additionally, Peer Educators (PE) were recruited from women’s self-help and community groups. ASHAs, ANMs, Anganwadi workers, and other PEs attended training sessions about reproductive health and cervical cancer, during which the training team used the educational flipbooks to lead didactic sessions, hold case discussions, role play, and review with gamification techniques. Prior to each screening camp, local PEs held education sessions using the flipbooks. Additional outreach was conducted by placement of fliers around the community.

*Screening facilities*

The screening programs were conducted in sites provided by the communities, including primary healthcare facilities, Anganwadi centers, schools, residences, and other locations that were accessible to all regardless of age, religion, or caste. Infrastructure varied greatly between the sites (Table 1). All necessary screening supplies and temporary infrastructure were brought to the screening site. Depending on-site needs, this included examination tables, light sources, backup power, privacy curtains, clean water, disinfectant, and all materials needed for pelvic examination. 

*Interview and examination*

Informed consent was obtained from all individual participants included in the study. Counselors administered a structured 10-minute questionnaire on socio-demographic characteristics, medical and reproductive history, and healthcare seeking practices. Participants underwent physical examination, including speculum examination with VIA. During VIA, if the entire squamocolumnar junction (SCJ) was visible, the examination was described as ‘adequate’; otherwise, it was described as ‘inadequate’. A positive VIA test was characterized by an opaque, dense, distinct acetowhite lesion with sharp margins on the cervix in close proximity to the SCJ, based upon the International Agency for Research on Cancer (IARC) 2003 guidelines (Sankaranarayanan and Wesley, 2003).

*Screening algorithm, laboratory investigation, and follow-up*

During the initial training period, women with adequate-positive VIA were offered biopsy and treatment with cryotherapy or LEEP according to the “screen and treat” methodology described by The Alliance for Cervical Cancer Prevention in 2004 (Sankaranarayanan, 2003). However, due to low acceptance of same-day treatment, the “screen and treat” method was discontinued, and a “screen, test, and treat” method was employed, with confirmatory Pap smears for women who were VIA adequate-positive or inadequate. Pap smears were classified based on the Bethesda system (Solomon and Nayar, 2004). Under both protocols, patients with adequate-negative VIA results were referred for rescreening in three years. Patients with normal laboratory results were informed over the phone or in person at follow-up visits. For patients with abnormal laboratory results, the team called by phone for women to come to the static clinic and met women who were not reachable in their communities. 

*Statistical analysis*

Data were entered in a Microsoft Excel database (Microsoft Corporation, Redmond, WA). Conventional descriptive statistical analyses were performed to analyze the characteristics of study participants. 

## Results

*Participant characteristics*

Over 50 PEs were recruited from over 30 communities, including women’s empowerment, occupational, and religious groups. In total, 190 camps were attended by 3821 registered participants. Of the registered women, 3544 (92.8%) were screened with VIA. Of the 277 women who were not screened, only 52 (18.8%) declined screening after enrollment. Other common reasons for VIA ineligibility included current vaginal bleeding (n=101, 36.5%); inability to visualize cervix (n = 24, 8.7%); prior hysterectomy (n=5, 1.8%); ineligible age on repeat questioning (n=4, 1.4%); or visible cancer (n=3, 1.08%). Women whose cervix was not visible and those with visible cancer were referred for follow-up at a collaborating hospital. Among women who were screened, the average age was 38.7 (SD: ±8.3) years. Nearly all women were ever married (99.9%), with the average years since marriage, an approximate of years since sexual debut, of 21.0 (SD: ±9.2) years. In total, 23 (0.6%) women reported having previously been screened for cervical cancer. Participants’ demographics and reproductive history are described in Table 2. 

*VIA results, follow-up, and treatment*

VIA was conducted for 3,544 women. The SCJ was adequately visualized in 2,752 (77.7%). Among all 3544 women, 12.4% (n=440) were VIA positive, and 169 (38.4%) of them received Pap smears (Table 3). The Pap smear showed atypia or dysplasia for 40 (23.7%) VIA positive women. In total, 699 VIA negative women underwent Pap smears. Among all VIA negative women who received Pap smears, 107 (15.4%) had atypia or dysplasia, including 12 women who had been characterized as having adequate-negative VIA. 

*Screen and treat*

**Figure 1:**
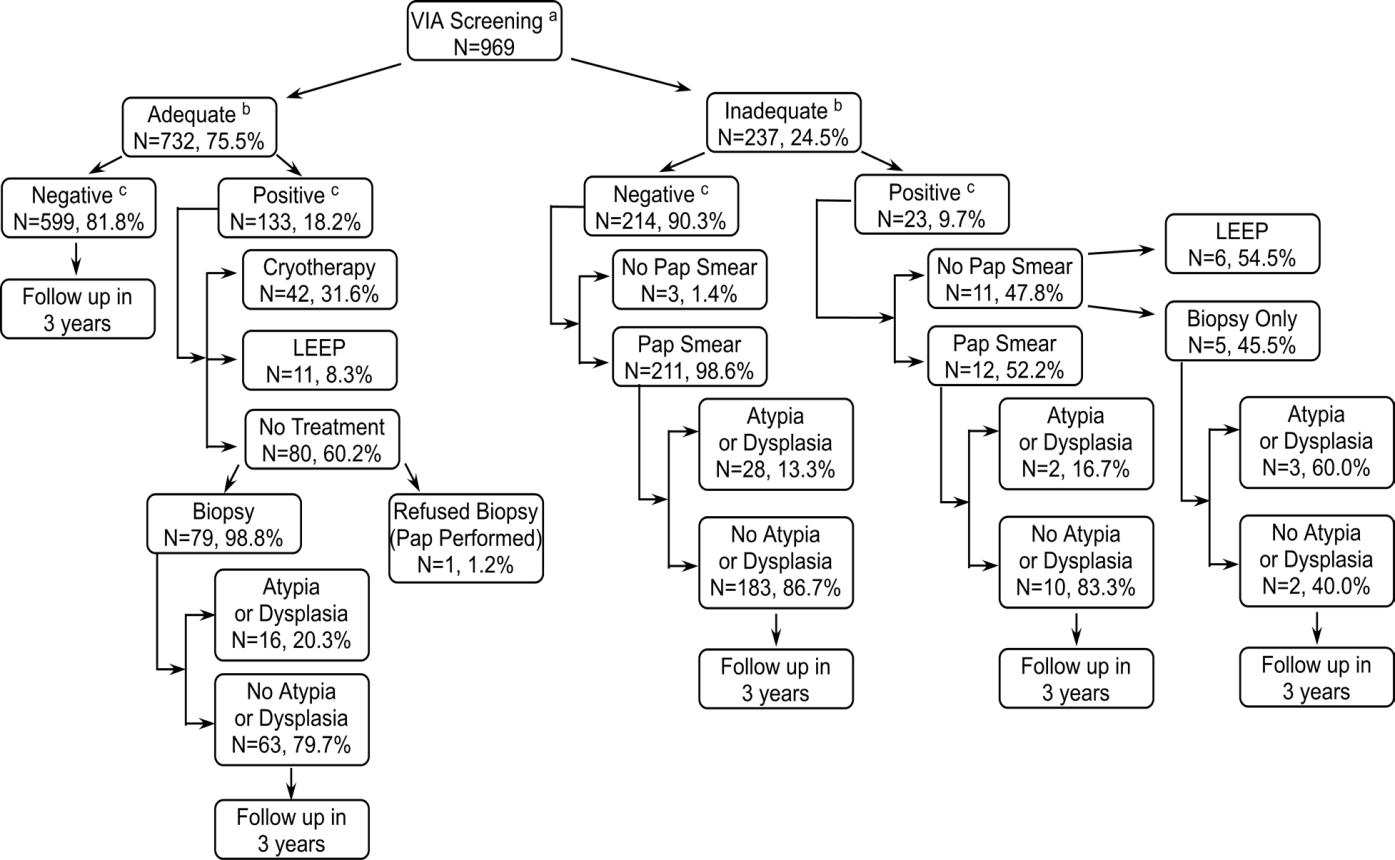
Results of a Community-Based Mobile Cervical Cancer Screening Program Using a “Screen and Treat” Visualization with Acetic Acid Protocol in Mysore, India, 2010-2018. a, Visual inspection with acetic acid; b, Adequate refers to the visualization of entire SCJ. Inadequate refer to incomplete visualization of SCJ; c,VIA positive indicates the presence of an opaque, dense, distinct acetowhite lesion with sharp margins on the cervix in close proximity to SCJ. VIA negative indicates the absence of such a lesion; Note, Some women received Pap smears and biopsies in addition to LEEP and cryosurgery

A total of 969 women were examined under the “screen and treat” protocol (Figure 1). Of 133 women with VIA adequate-positive lesions, only 49 (36.8%) received same-day treatment with LEEP (n=7, 5.3%) or cryotherapy (n=42, 31.6%). An additional four (3.0%) women returned for LEEP at a follow-up visit. All women with adequate-positive lesions who did not undergo treatment (n=80) had confirmatory testing, and 17 (21.3%) had atypia or dysplasia. Additionally, of the 237 (24.5%) women with inadequate VIA examinations, 93.7% (n=223) received Pap smears, and 13.5% (n=30) had atypia or dysplasia. On follow-up, of all 969 women, an additional seven (0.7%) women went on to receive LEEP (n=5, 0.5%) or cryotherapy (n=2, 0.2%). Anecdotally, providers reported common patient-related reasons for refusing treatment included wanting to consult with another doctor or family member and concern for violence if they refused sex, as pelvic rest was recommended post-treatment. Provider-related reasons included deferral to another camp day if they were unsure about VIA positivity, especially with concurrent cervicitis. Due to the low treatment rate, the program transitioned to a “screen, test, and treat” protocol.

*Screen, test, and treat*

**Figure 2:**
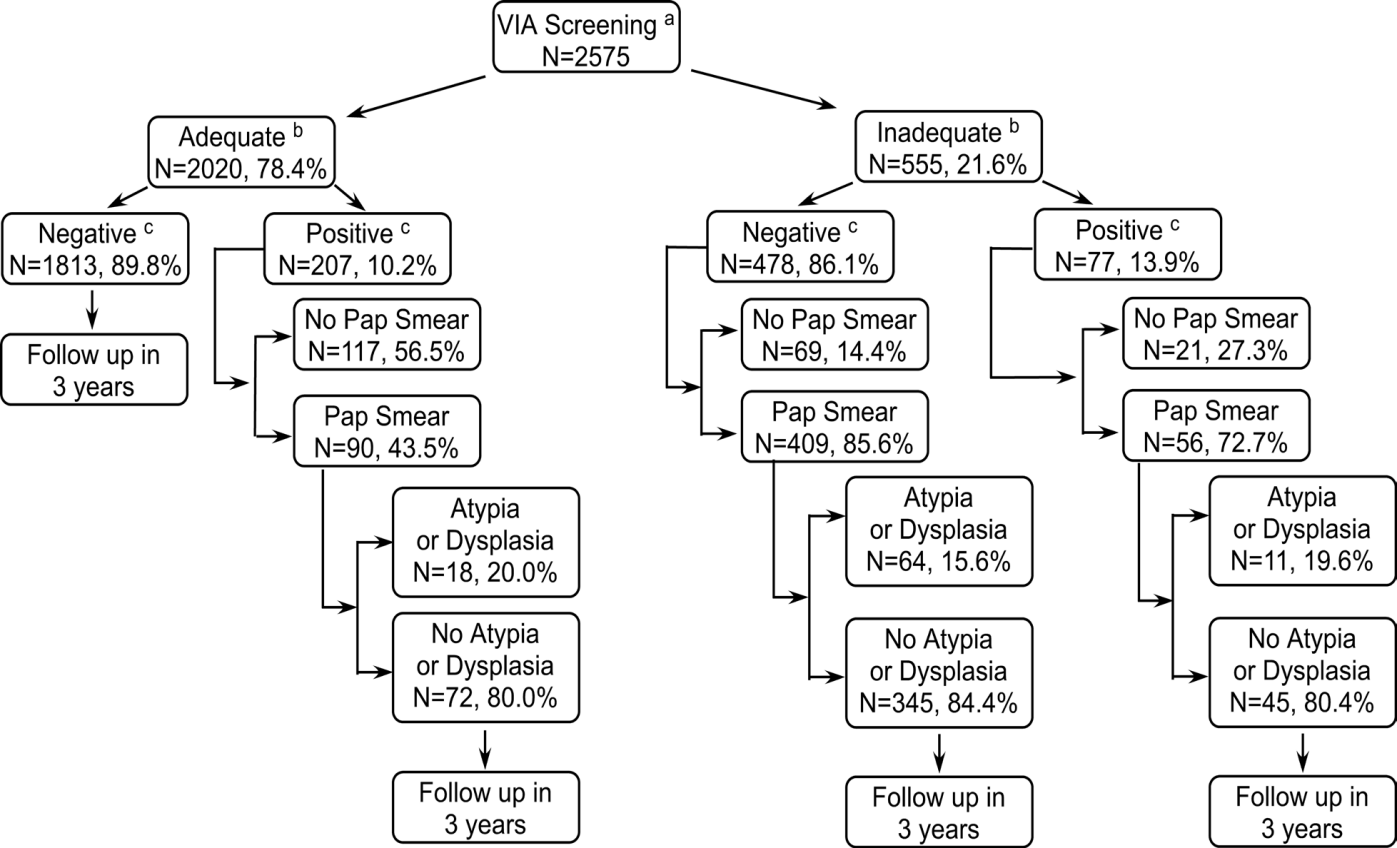
Results of a community-based mobile cervical cancer screening program using a “screen, test, and treat” visualization with acetic acid protocol in Mysore, India, 2010-2018. a, Visual inspection with acetic acid; b, Adequate refers to the visualization of entire SCJ. Inadequate refer to incomplete visualization of SCJ; c, VIA positive indicates the presence of an opaque, dense, distinct acetowhite lesion with sharp margins on the cervix in close proximity to SCJ. VIA negative indicates the absence of such a lesion

Under the “screen, test, and treat” protocol, 2575 women underwent VIA (Figure 2). Of those, 762 were eligible for follow-up Pap smear, of whom 555 (72.8%) received one. Among 207 adequate-positive VIA cases, 43% (n=90) received Pap smears, and 18 (20%) had atypia or dysplasia. Of the 555 (21.6%) women with inadequate VIA examinations, 465 (83.8%) received a Pap smear, and 16.3% (n= 76) had atypia or dysplasia. Of note, the first year of the “screen, test, and treat” program overlapped with provider training, and Pap smears were deferred to training sessions. However, no eligible participants returned for Pap smears. After training was completed, same-day Pap smears were offered at all “screen, test, and treat” clinics. During that period, 465/495 (93.4%) of women with inadequate results and 90/138 (65.2%) with adequate-positive results received Pap smears. Anecdotally, providers shared that they did not perform Pap smears for the same reasons why VIA exams were inadequate – if cervices were inaccessible or if participants began bleeding during the examination. These women were referred for follow-up care at collaborating hospitals.

*Biopsies*

In total, 190 women received biopsies, including prior to cryotherapy, during LEEP, for polyps or other lesions, and some women with new VIA positive lesions on follow-up. Biopsy reports were available for 179/190 (94.2%) women. However, 14.5% (n=26) samples were inadequate for interpretation. Of the 153 women with sufficient samples, 63 (41.2%) had atypia or dysplasia, including 24 (15.7%) with CIN2+ lesions. Women with atypia or dysplasia on biopsy were referred to a tertiary care center, and 27 of them (42.9%) received treatment during a camp with cryotherapy (n=19) or LEEP (n=8). Of the 24 women with CIN2+ lesions, two-thirds (n=16, 66.7%) received treatment during a camp with cryotherapy (n=9) or LEEP (n=7). Among all VIA positive women, 134 had biopsy reports with sufficient samples, 62 (46.3%) of whom had atypia or dysplasia. 

*Treatment follow-up*

Overall, 56 women underwent cryotherapy and 27 received LEEP. Of these 83 women, 49 (59.0%) were followed-up regularly at the static clinic. All 49 women were VIA-negative on repeat examination between 6 months and 2 years of treatment. Additionally, 10 women underwent hysterectomy.

**Table 1:** Characteristics of Community-Based Mobile Cervical Cancer Screening Sites in Mysore, India, 2010-2018 (N=58)

Infrastructure	Assessment of facility components
Electricity	All sites had intermittent electricity supply
Lighting	Most sites did not have lighting. The clinic team brought battery-operated headlamps for patient examination.
Water supply	Occasionally sites did not have running water. Community members transported water to the screening site.
Examination room	In non-clinical spaces, the team covered all doors and windows to provide adequate privacy.
Patient counseling room	When screening sites did not have a room for patient counseling, the counseling team identified a separate private location.
Examination table	Screening camps located in Primary Health Centers were equipped with examination tables. For community-based sites, the clinic team provided custom foldable stainless steel pelvic examination tables.
Supplies for pelvic examination, VIA screening and Pap smear preparation	All screening supplies were brought to screening sites by the clinic team.
Staff	
Staff workload	Physicians conducted the initial training camps, after which nurses screened under the supervision of one on-site doctor .

Note: Table- 1 was adapted from Shiferaw, N., Salvador-Davila G, Kassahun K, et al. 2016

**Table 2:** Demographic and Reproductive History of Women Screened at a Community-Based Mobile Cervical Cancer Screening Program in Mysore, India, 2010-2018 (N=3,544)

Characteristic	N (%)
Mean current age in years (SD)	38.7 (8.3)
HIV status	
Never tested	1191 (33.6)
Negative	2286 (64.5)
Positive	67 (1.9)
Mean years of marriage (SD)	21.0 (9.2)
Contraception methods	
Female sterilization	2437 (68.76)
Condoms	53 (1.5)
Copper intra-uterine device	30 (0.9)
Oral tablets	17 (0.5)
Vasectomy	12 (0.3)
Withdrawal	10 (0.3)
All others	8 (0.2)
None	979 (27.6)
Post-menopausal	
Yes	697 (19.7)
No	2847 (80.3)
Gravidity	
0	98 (2.8)
1	221 (6.2)
2	1383 (39.0)
3 or more	1837 (51.8)
Prior cervical cancer screening	
None	3521 (99.4)
VIA	13 (0.4)
Pap smear	10 (0.3)

SD, standard deviation; Post-menopausal, as defined by the participant

**Table 3:** Results of Visual Inspection with Acetic Acid and Pap Smear at a Community-Based Mobile Cervical Cancer Screening Program in Mysore, India, 2010-2018

Year	No. women screened	No. VIA positive women	No. VIA positive women who received Pap smear	No. VIA positive women with atypia or dysplasia on Pap smear
2010-2012 (ST)	969	156 (16.1%)	23 (14.74%)	6 (26.1%)
2011-2012 (STT)	227	85 (37.4)	0 (-)	0 (-)
2013 (STT)	243	14 (5.8%)	4 (28.57%)	2 (50%)
2014 (STT)	950	47 (4.9%)	27 (57.45%)	11 (40.7%)
2015 (STT)	694	58 (8.4%)	54 (93.1%)	10 (18.5%)
2016 (STT)	175	38 (21.7%)	22 (57.89%)	2 (9.1%)
2017 (STT)	83	11 (13.3%)	10 (90.91%)	4 (40%)
2018 (STT)	203	31 (15.3%)	29 (93.55%)	5 (17.2%)
Total	3544	440 (12.4%)	169 (38.41%)	40 (23.7%)

VIA, Visual Inspection with Acetic Acid; ST, Screen and Treat protocol; STT, Screen, Test, and Treat protocol

## Discussion

Barriers to cervical cancer screening in rural areas of Mysore district include low awareness, lack of infrastructure and human resources for screening, and distance to centralized healthcare facilities. However, we were able to turn these challenges into programmatic strengths through community engagement, mobile infrastructure, and task-shifting. By gaining buy-in from community leaders, we brought screening to 58 public spaces accessible regardless of caste or religion. Furthermore, by working with existing social networks and groups, we identified and trained over 50 enthusiastic PEs who led multiple education sessions in their communities. Early camp-based screening in rural India without education and outreach resulted in low acceptance of screening (Desai, 2004). In contrast, our high rates of acceptance are in line with previous studies demonstrating increased uptake of screening with community-based education programs (Musa et al., 2017).

While community-based settings lack the resources available at a clinic or hospital, we were able to utilize established resources by consulting community leaders, scouting locations, and adapting our mobile infrastructure for each site. This was a key step, as travel can be a significant barrier for women to access screening, even when they do not have to pay for it (Gravitt et al., 2010; Montgomery et al., 2015). Our experience demonstrates that schools and Anganwadi buildings can be used for screening and provides a model for infrastructure considerations when planning for screening camps as part of the expansion of screening under the Indian Government’s 2016 recommendations. 

Finally, as we utilized task shifting as rural Medical Officers in India report difficulty managing high case loads, and there is demand for additional human resources (Vallikunnu et al., 2014). In systems with a shortage of healthcare providers, task shifting is a key tool to provide high quality, cost-effective, accessible care (Mezei et al., 2017). In our program, outreach, counseling, and explanation of screening procedures were performed by PEs and outreach workers. Furthermore, the majority of screenings were performed by nurses under the supervision of a single doctor. This model allowed for a larger number of women to be screened without creating undue burden on rural Medical Officers.

Despite these successes, there are ongoing challenges and room for improvement for future community-based cervical cancer screening projects, namely improving “screen and treat” uptake, minimizing loss to follow-up, and improving test accuracy.

A major limitation of our program was the low uptake of the “screen and treat” approach, with less than half of eligible women agreeing to receive same-day treatment. As the “screen and treat” method has long been considered highly acceptable around the world, including in India (Mandal and Basu, 2018), further investigation is warranted into why this population had low acceptance and how best to combat it. One earlier study in Andhra Pradesh, India suggested that the perception that treatment is not needed for asymptomatic conditions was an important factor limiting acceptance of treatment (Gravitt et al., 2010). Increased acceptance may be encouraged by further education targeting this perception as well as possible treatment outcomes. Additionally, given our participants’ concerns regarding negotiating pelvic rest with their sexual partners, it may be beneficial to make more effort to include men in education and outreach.

One of the largest challenges in our project was significant loss to confirmatory testing, treatment, and post-treatment follow-up. In the first few years of the program, some VIA positive women were given referrals or instructed to return. However, as the high rate of loss to follow-up became clear, greater emphasis was put on counseling for same-day confirmatory testing. From 2014 onwards, the majority of VIA positive women received pap smear on the same visit (Table 3). The increased uptake demonstrates that offering same-day services and improving counseling can improve retention for confirmatory testing. Additionally, prior studies have suggested improving follow-up by incorporating community-based health advocates, coordinating local stakeholders, and systematically tracking patients (Moon et al., 2012; Paul et al., 2013; Shiferaw et al., 2016). We attempted to implement these strategies, including calling screened women to visit the static clinic in Mysore city. However, visiting the clinic for treatment may present the same barriers that prevent screening – inability to travel, miss work, lack of understanding, and fear (Karunakaran et al., 2017). Therefore, there is a need to bring not only screening, but also follow-up care directly to women when possible. In the future, ‘screen, test, and treat’ campaigns may be more successful if they coordinate follow-up camps to explain results and offer confirmatory testing and treatment. 

Lastly, when patients have limited access to follow-up care, the ‘screen and treat’ approach using VIA can be lifesaving. However, our data from the “screen, test, and treat” program demonstrated that only 23.3% of women who were VIA positive had atypia or dysplasia on Pap smear. Therefore, a VIA-based “screen and treat” approach would have over-treated 76.7% of women. Screening tests are intentionally designed to have relatively high sensitivity at the cost of lower specificity. However, that is on the premise that follow-up for diagnosis and treatments are available and acceptable(Organization, 2010). Among our participants, follow-up testing was a challenge, and even with additional steps as outlined above, uptake of treatment remained limited. Furthermore, false positives on screening tests are associated with additional psychological burden, complications from testing and treatment, and higher costs (Maxim et al., 2014). Among our patients, the social impact of VIA positivity included discussion of gynecologic disease and pelvic rest with family members who could be violent. Taken together, our experience with nine years of VIA-based screening suggest that other screening techniques may be more useful in the rural Mysore setting. For example, screening with self-collected vaginal swabs for HPV DNA testing would dramatically reduce the number of women who need pelvic examination, requiring tracking for follow-up testing, and ultimately undergo treatment. 

The VIA screening program demonstrates that mass screening of rural women in non-clinical settings is possible through extensive community outreach and use of mobile infrastructure. This is a crucial strategy to increase the current unacceptably low rates of screening in India, until costlier screening technologies such as HPV DNA testing or cervical cytology become widely available. Future research should examine how to increase linkage to treatment for VIA positive women, including improving acceptance of single visit “screen and treat” methods and uptake of follow-up for “screen, test, and treat” protocols.

## Author Contribution Statement

Study conception and design: Vijaya Srinivas, Sasha Herbst de Cortina and Purnima Madhivanan; Data collection: Vijaya Srinivas, Purnima Jayakrishna,, Kavitha Ravi, Anisa Khan; Analysis and interpretation of results: Holly Nishimura, Sasha Herbst de Cortina, Vijaya Srinivas; Draft manuscript preparation and preparation of flowcharts: Vijaya Srinivas, Sasha Herbst de Cortina Karl Krupp, Purnima Madhivanan, SubbaRao V. Madhunapantula; All authors reviewed the results and approved the final version of the manuscript 
